# Empowering Radiographers: A Call for Integrated AI Training in University Curricula

**DOI:** 10.1155/2024/7001343

**Published:** 2024-03-08

**Authors:** Mohammad A. Rawashdeh, Sara Almazrouei, Maha Zaitoun, Praveen Kumar, Charbel Saade

**Affiliations:** ^1^Faculty of Health Sciences, Gulf Medical University, Ajman, UAE; ^2^Faculty of Applied Medical Sciences, Jordan University of Science and Technology, Irbid 222110, Jordan; ^3^Department of Diagnostic Radiography, UG 12 Aras Watson, Brookfield Health Sciences, T12 AK54, University College Cork, Cork, Ireland

## Abstract

**Background:**

Artificial intelligence (AI) applications are rapidly advancing in the field of medical imaging. This study is aimed at investigating the perception and knowledge of radiographers towards artificial intelligence.

**Methods:**

An online survey employing Google Forms consisting of 20 questions regarding the radiographers' perception of AI. The questionnaire was divided into two parts. The first part consisted of demographic information as well as whether the participants think AI should be part of medical training, their previous knowledge of the technologies used in AI, and whether they prefer to receive training on AI. The second part of the questionnaire consisted of two fields. The first one consisted of 16 questions regarding radiographers' perception of AI applications in radiology. Descriptive analysis and logistic regression analysis were used to evaluate the effect of gender on the items of the questionnaire.

**Results:**

Familiarity with AI was low, with only 52 out of 100 respondents (52%) reporting good familiarity with AI. Many participants considered AI useful in the medical field (74%). The findings of the study demonstrate that nearly most of the participants (98%) believed that AI should be integrated into university education, with 87% of the respondents preferring to receive training on AI, with some already having prior knowledge of AI used in technologies. The logistic regression analysis indicated a significant association between male gender and experience within the range of 23-27 years with the degree of familiarity with AI technology, exhibiting respective odds ratios of 1.89 (COR = 1.89) and 1.87 (COR = 1.87).

**Conclusions:**

This study suggests that medical practices have a favorable attitude towards AI in the radiology field. Most participants surveyed believed that AI should be part of radiography education. AI training programs for undergraduate and postgraduate radiographers may be necessary to prepare them for AI tools in radiology development.

## 1. Introduction

Artificial intelligence (AI) is rapidly expanding in the field of computing and informatics, with high applicability to medicine [1]. In the healthcare sector, AI has become a key component of different applications, including remote patient monitoring, drug discovery, imaging, and medical diagnostics, wearables, risk management, hospital management, and virtual assistants. Several areas with big data components, such as ribonucleic acid (RNA) and deoxynucleic acid (DNA) sequencing data analysis [2], are also expected to benefit from AI algorithms to solve complex and time-consuming tasks. Moreover, medical fields relying on imaging data, such as dermatology [3], radiology, pathology, and ophthalmology [4], have now started to take advantage of AI method implementation in their routine clinical environments. In radiology, trained physicians assess and visually report findings and medical images to detect, monitor, and characterize diseases. This kind of assessment is usually based on experience and education, and it can be subjective. Unlike such qualitative reasoning, AI excels in recognizing complex patterns in imaging data and can robotically deliver a quantitative valuation. Integrating AI into clinical processes as a tool for assisting radiologists and physicians generally, it can achieve more reproducible and accurate radiology assessments.

Previously, there was a belief that AI could replace physicians in various specialties [5–8]. However, this has not come to pass, as current scientific developments have been quick to reinforce such thoughts. IBM's Watson was established from an extensive database of medical records and published literature [9, 10], which has allowed AI to contribute to the formation of proper treatment and precise diagnosis plans [9, 10]. Additionally, Watson offers advice on top cancer treatments and performs genome analyses [11]. Recently, AI has been employed to predict genetic differences in low-grade gliomas [12], reduce false-positive rates in computer-aided screening mammography detection [13], recognize genetic phenotypes within small-cell lung carcinoma [14], conduct automatic bone age assessments [15], and enhance the detection of pathologic mediastinal lymph nodes [16]. Moreover, Google's DeepMind software is being employed to test the feasibility of automated grading of digital fundus photographs by optical coherence tomography [17]. The previous examples illustrate the impact of AI in medicine. In the future, AI applications will likely be extended to other fields, leading to significant changes in the role of physicians and the way they practice medicine [15].

A qualitative survey of radiographers' perspectives on AI in Australia has reported high-priority attitudes towards automated complex tasks in image quantitation, segmentation, and reconstruction, as well as improving image quality by dose/noise reduction and pseudo-CT for attenuation correction [18]. While Australian radiographers and nuclear medicine technologists, along with their clinical departments, are not yet fully prepared for AI implementation, the survey also revealed a strong desire to acquire the necessary knowledge and skills for clinical development [18]. In contrast, radiographer perspectives on AI in Africa indicate a lack of skill bases, education, and awareness surrounding AI in the workforce. Similarly, they lack the foundation to understand the application of AI to their profession but are open to its potential to improve. A UK study reported that education would benefit their careers and that training should be provided [19]. To our knowledge, no similar study has been conducted in the Middle East. Through this study, we will investigate radiographers' perceptions and attitudes towards AI implementation in the medical sector and radiology.

## 2. Materials and Methods

An ethical application was submitted to Gulf Medical University in the United Arab Emirates and approval was granted. The institutional review board waived the need for participants to provide consent for this descriptive study. Informed consent for participation was also waived, but participants provided their informed consent before answering the questionnaire. In this study, radiographers were asked to rate their knowledge, attitude, and perception towards the implementation of AI applications in the medical imaging field.

### 2.1. Participants

Data were collected via an online survey (Google Survey) that was distributed through social media networks. No identifying personal information was collected. Participants were recruited using nonprobability sampling techniques, including convenience and snowball sampling. Based on Cohen's formula (1992), a power analysis showed that a sample size of 100 would provide an 80% chance of detecting correlations with a significance level of *p* ≤ 0.05.

### 2.2. Questionnaire

The questionnaire used in this study was adapted from Oh et al. and Pinto Dos Santos et al. [20, 21], with minor modifications made to the original surveys, including changes to the phrasing of some questions and the addition of options for certain questions. The questionnaire consisted of two sections: personal information, including gender, age, specialty, experience, workplace, country, and self-assessment of familiarity with AI. The personal information section also included questions on whether participants believed that AI should be part of medical training, their prior knowledge of the technologies used in AI, and whether they would prefer to receive training on AI.

The second part of the questionnaire consisted of two fields; the first one consisted of 14 questions regarding radiographers' perception of the medical field in general, and the second field consisted of two questions regarding the radiographer's professional perception of AI applications in radiology. The 16 questions included multiple-choice questions, true/false questions, and a 5-point Likert scale besides open-ended questions. In general, the questions aimed at testing the attitude towards AI, the expected applications of AI in medicine, and possible risks and opportunities of AI in the medical fields and radiology.

As mentioned above, the questions used in this study are based on previous literature, with some modifications made by adding or excluding certain questions. To validate these changes, the updated questionnaire was presented to a panel of experienced information technology lecturers and medical professionals who were not included in the study sample. The study tool was also validated by a group of 10 medical professionals who assessed the questionnaire for clarity. These doctors were subsequently excluded from the study sample. The reliability of the study tool was confirmed using the test-retest method, whereby the pilot study was readministered to the same sample of 10 radiographers within two weeks.

### 2.3. Data Analysis

For analysis of data, means, standard deviation, frequencies, and percentages were extracted for each question using the SPSS program. Univariate logistic regression was employed to evaluate the autonomous determinants influencing the extent of acquaintance with AI technology, considering various associated factors. Crude odds ratios (CORs) were calculated along with their corresponding 95% confidence intervals (CIs). All significance tests were two-sided with a *p* value less than 0.05 as an indicator of statistical significance.

## 3. Results

A total of 100 participants were recruited for this study. The survey was conducted with both male and female participants, with a gender split that was largely reflective of the workforce in the United Arab Emirates. The majority of respondents were male (58.2%) and the remainder were female (41.2%). The age range of participants varied, with the highest percentage of radiographers being aged 23-27 years (37.7%), followed by 28-32 years (19.7%). The lowest percentage of radiographers were in the age range of 38-42 years (8.3%) and 18-22 years (8.9%). The study included participants from various clinical settings, including university hospitals (43.8%), government hospitals (32.1%), private clinics (14.4%), and military hospitals (9.7%). Most participants had experience in medical practice, with 57.8% having fewer than five years of experience and 19.7% having more than six years of clinical practice. The study also included 22.5% of participants without clinical expertise (Table 1).

A notable disparity was observed in the levels of agreement and disagreement in response to the statement, “Do you agree that you have a good familiarity with artificial intelligence?” Specifically, 52 radiographers concurred that they possess good familiarity with artificial intelligence. The majority of the radiographers express a belief in the beneficial applications of artificial intelligence in the field of radiology. Conversely, a lower percentage of respondents agreed that AI surpasses doctors in the medical field concerning diagnostic ability. Nevertheless, half of the participants reported incorporating AI into their medical diagnostic processes to enhance their capabilities in practice, and this difference was not deemed statistically significant.  Table 2 provides further details.

The study's findings reveal that nearly all participants (98%) endorsed the integration of AI into university education. Moreover, 87% of respondents expressed a preference for receiving AI training, with some already possessing prior knowledge of AI applications in technologies. Approximately one-third of participants (32%) reported having prior knowledge of the technologies employed in AI. When queried about the transformative potential of AI in radiology, only a minority of participants (34%) acknowledged its capacity to revolutionize the field (see Table 3).

Most of the participants seek information on AI through online sources or happen upon it by chance. The Internet and media were the primary sources of information on AI, accounting for 59% of the responses, followed by colleagues and friends at 13%. Books and journals accounted for only 10% of the responses, with other sources contributing to 4%. Regarding the role that AI could play, a majority of the participants believe that it could have a positive impact. Specifically, 50% of the participants think that AI could be used to aid in some cases, 36% of the respondents believe that it could form the basis of evidence-based medical care, 35% believe that it could fill in gaps created by the limitations of human intelligence, and 9% believe that it could replace a doctor's judgment. However, a minority of the participants (18%) believed that AI would not positively impact the health industry and would be of no help in diagnosis or treatment. When evaluating the diagnostic capabilities of AI, 36% of respondents deemed it inferior compared to a doctor. However, more than half of the participants acknowledged that AI was at par with, if not superior to, a doctor. Specifically, 18% of respondents believed that it was at par, 26% thought it was slightly superior, and 6% indicated that it was far superior to a doctor's experience. Although the majority of participants deemed AI's capabilities to be at par with or better than a radiographer's experience, only a few would choose AI's judgment (13%) or seek out other AI judgments (6%) in case of a disagreement. The majority indicated that they would seek an expert's judgment (41%), follow their judgment (17%), or bring up the issue with the patient and let them have the final say (9%) (Table 4).

The logistic regression analysis indicated a significant association between male gender and experience within the range of 23-27 years with the degree of familiarity with AI technology, exhibiting respective odds ratios of 1.89 (COR = 1.89) and 1.87 (COR = 1.87). No other statistically significant predictors were identified, as presented in Table 5.

## 4. Discussion

The study investigated radiographers' perceptions of AI transformation in medical imaging in the United Arab Emirates. The majority of respondents expressed a desire to receive AI training in medical imaging. The lack of AI in medical imaging was acknowledged, and the majority of participants (59%) relied on the Internet and social media for information on the topic. There is a need to address this gap in knowledge by incorporating educational training on AI into radiography education for under or postgraduate radiographers in UAE medical universities and colleges. In response to this need, the City University London has introduced a radiographer-specific AI course, reflecting the growing awareness of the importance of AI knowledge in the medical field. Postgraduate courses are also beginning to integrate AI elements where appropriate, such as a section on “the use of AI in image interpretation/reporting” in postgraduate radiographer reporting courses.

The study reveals that many have some background knowledge of AI, consistent with previous studies from Ghana, where 86.1% of participants expressed awareness of AI in medical imaging practice, and Saudi Arabia, where 83% of radiologists were aware of the concept of AI in machines. Despite being a newly introduced study in medical imaging, African study participants were aware of AI and had positive expectations for its potential to change traditional modes of medical imaging practice 18, 19.

However, participants also expressed concerns that AI tools may lead to diagnostic errors due to associated margins of error with mechanical systems. AI combined with radiographer double reading of imaging examinations may provide an opportunity to measure the potential for radiographer-led reporting backlogs and workforce. While AI is a critical tool for assisting in the medical field, some study participants had reservations about whether AI is superior to doctors' experience in diagnostic ability. Based on the results of the experiment, 41% of participants had abandoned the idea of seeking an AI's judgement and preferred to rely on an expert's judgement instead.

In addition, there is a notable psychological concern that patients and healthcare professionals may experience fear and mistrust due to the limited capacity of existing AI systems to be explained and their lack of perceptual ability. The previous study showed that the introduction of AI to universities may be misunderstood and cause fear in students that AI will overcome radiologists, leading them to believe that they should not enter this medical field [22]. Radiographer participants in the current study expressed that artificial intelligence has useful applications in the medical field and would lead to innovations and growth in medical imaging practice, including nuclear medicine. This can be attributed to the limited information on AI available to them, mainly through media and the Internet. Although almost half of the respondents would prefer the inclusion of AI into medical services, information and accessibility to AI are still lacking in the sector. The findings also show that a significant percentage of participants rely on the Internet and media as the primary source of information about AI, highlighting how the current technological evolution and social media have revolutionized knowledge and practice.

Gender and age within the 23 to 27 range were recognized as potential factors linked to the familiarity level with AI technology. Our study uncovered a notable association between the male gender and less-experienced radiographers, demonstrating odds ratios of 1.9 and 1.89, respectively, with familiarity with AI technology. This outcome might be attributed to increased apprehension regarding new AI technology among both older practitioners and females.

Limitations of the study include the small sample size of radiographers practicing in the United Arab Emirates, which may not be representative of the worldwide radiographer population with variations in educational provisions, clinical practice, and roles within radiography. Another limitation is that the demographic information was self-reported, and there was no way to verify the accuracy of the information provided by the participants. This may have led to inaccurate or incomplete information and could affect the generalizability of the findings. Finally, there was no determination which centers were employing AI routinely compared to those that have an interest in it with no AI in their workflow.

In conclusion, the study found that the majority of radiographers surveyed in the United Arab Emirates believed that artificial intelligence has useful applications in medical imaging, including reducing workload, improving efficiency, and reducing medical errors. The study suggests that AI training programs for undergraduate and postgraduate radiographers may be necessary to prepare them for AI tools in radiology development. Despite some participants expressing concerns about AI adaptation threatening their jobs, the study demonstrated a high level of motivation among participants to learn and incorporate AI concepts into their clinical practice with proper education and training programs. The study highlights the need for increased accessibility and information on AI in medical and university education.

## Figures and Tables

**Table 1:** Demographic details of respondents.

Participant gender	Female	41.8%
	Male	58.2%

Age range (%)	18-22 years	8.9%
	23-27 years	37.7%
	28-32 years	19.7%
	38-42 years	8.3%

Clinical setting/counts	University hospital	43.8%
	Governmental hospital	32.1%
	Private clinic	14.4%
	Military hospital	9.7%

Years practicing radiography	No experience	22.5%
	Less than 5	57.8%
	More than 6	19.7%

**Table 2:** Level of familiarity with the AI technology.

Item	Statement	Radiographer (agree)	
		*N*	%
1	Do you agree that you have good familiarity with artificial intelligence?	52	52%
2	Do you agree that artificial intelligence has useful applications in the radiology field?	74	74%
3	Do you agree that the diagnostic ability of AI is superior to the clinical experience of a human doctor?	40	40%
4	Do you agree that you would always use AI when making medical decisions in the future?	50	50%

**Table 3:** Education and training received by radiographers.

Item	Statement	Radiographer (yes)	
		*N*	%
1	Do you think that artificial intelligence should be a part of university training?	98	98%
2	Do you have a previous knowledge of the used technologies in AI?	32	32%
3	Do you prefer to receive training on AI?	87	87%
4	Artificial intelligence has the ability to revolutionize radiology	34	34%

**Table 4:** Perceptions of radiographers towards artificial intelligence and source of knowledge.

Question	Item	Statement	Radiographers	
			*N*	%
What is the source of your information about AI?	1	Internet and media	59	59%
	2	Books and journals	10	10%
	3	Colleagues and friends	13	13%
	4	Other sources	4	4%

What is the role AI could play in your practice? (You can select more than one response)	1	It will provide no help in the field of medical diagnosis/treatment	18	18%
	2	It will guide me in particular cases	50	50%
	3	It will be an evidence-based medical care basis	36	36%
	4	It will fill the gaps caused by human intellectual ability limits	35	35%
	5	AI will replace doctor's judgment	9	9%
	6	Other sources	4	4%

How do you evaluate the AI's diagnostic ability compared with the doctor's experience?	1	Less than the doctor's experience	10	10%
	2	Not superior	26	26%
	3	Similar	18	18%
	4	Superior	26	26%
	5	Highly superior	6	6%

If you have an AI, which you follow if there was a contradiction between your judgment and the AI's judgment?	1	My judgment	17	17%
	2	AI's judgment	13	13%
	3	Seek for the experts' judgment	41	41%
	4	Seek for other AI programs' judgment	6	6%
	5	Leave the decision to the patient	9	9%

**Table 5:** Logistic regression analysis of independent predictors for the level of familiarity with AI technology.

Variables	*p* value	COR	95% CI for OR	
			Lower	Upper
Gender				
Female	Reference category			
Male	0.042	1.89	1.024	3.53

Age range (years)				
18-22	Reference category			
23-27	0.462	1.79	0.38	8.43
28-32	0.362	1.39	0.48	4.21
38-42	0.234	1.721	0.61	9.78

Clinical setting/counts				
Governmental hospital	Reference category			
University hospital	0.040	1.87	0.89	2.03
Private clinic	0.060	1.47	0.22	1.22
Military hospital	0.201	1.51	0.80	2.81

Years practicing radiography (years)				
No experience	Reference category			
Less than 5	0.077	1.12	0.750	2.55
More than 6	0.241	1.52	0.27	2.82

COR = crude odds ratio; OR = estimated odds ratio; 95% CI = 95% confidence interval.

## Data Availability

The data used to support the findings of this study are available from the corresponding author upon request.
